# Omega-3 and Sports: Focus on Inflammation

**DOI:** 10.3390/life14101315

**Published:** 2024-10-16

**Authors:** Roberto Cannataro, Diana Marisol Abrego-Guandique, Natascia Straface, Erika Cione

**Affiliations:** 1 Galascreen Laboratories, University of Calabria, 87036 Rende, Italy; straface.natascia@gmail.com; 2 Research Division, Dynamical Business & Science Society—DBSS International SAS, Bogotá 110311, Colombia; 3 Department of Health Sciences, University of Magna Graecia Catanzaro, 88100 Catanzaro, Italy; dianamarisol.abregoguandique@unicz.it; 4 Department of Pharmacy, Health and Nutritional Sciences, University of Calabria, 87036 Rende, Italy

**Keywords:** omega-3 fatty acid, fish oil, inflammation, resolvins

## Abstract

Inflammation is expected in sports, especially when practiced at a high level. The human body is pushed toward its limit, and this is perceived as a “stressogenic agent”. Athletes, especially elite ones, desire it because their bodies can react with super-compensation, i.e., improve muscle mass, strength, speed, resistance, and, therefore, athletic performance. Thus, the inflammatory stimuli should be there during training but also counteracted to have the body placed in the optimal conditions for reacting with super-compensation. In this sense, omega-3 fatty acids have been shown to have anti-inflammatory biochemical activity. In this review, we will present the biochemical mechanisms of action of omega-3 fatty acids through their mediators, specialized pro-resolving mediators, which have anti-inflammatory activity. A focus will be on studies on omega-3 fatty acid supplementation in sports, and we will provide indications for possible practical applications and future studies, which are undoubtedly necessary to clarify the omega-3 fatty acids used in sports practice.

## 1. Introduction

The use of oral nutritional supplements (ONSs) is a widespread phenomenon, as summarized recently in a systematic review and meta-analysis [1]. Sometimes, their use can be harmful [2]. Despite this, it is common to notice that ONSs are often largely consumed by sport/physical activity practitioners [3]. Depending on the type of sport/physical activity practiced, it should be distinguished whether an ONS is used as a supplement to have an ergogenic effect (i.e., improve performance), for the rehabilitation of exercise-induced musculoskeletal injuries to repristinate muscle health, or to prevent possible stressful conditions [4]. When the human body is subjected to repeated chronic stressors (such as hypo-energetic conditions or sleep deprivation and extensive training), the normal physiology/biology and its biochemical mechanisms operate outside their usual ranges. In the case of extensive training, this causes a suffering condition, triggering inflammation. It is worth noting that there exists an ordinated biochemical process to prevent the progression from acute non-resolved inflammation to chronic persistent inflammation. When it is not possible to reach a homeostatic condition again, the human body starts a process of adaptation. This adaptation, of course, has a cost in terms of energy for our body, and it is known as allostatic load [5]. In exercise sciences, this adaptation process is resumed by the concept of super-compensation. This suggests that following a stressor (such as resistance workload), there is a need for energy (nutrition) and time (rest) to compensate for the decline in performance. This facilitates muscle strength and/or mass enhancement [6]. Keeping the system in the right conditions should result in functional overreaching (FOR), leading to positive adaptations (e.g., greater muscle strength or better aerobic capacity). However, if these conditions are not met, the organism experiences non-FOR, leading to a decline in performance that, if prolonged over time, can give a pseudopathological state known as overtraining syndrome [7]. In this sense, inflammation is a double-edged sword. It is necessary to trigger the regenerative processes that lead to FOR [8], but it must be modulated; otherwise, it could be negative for performance. In this review, in particular, the molecules synthesized from omega-3 fatty acids, categorized as “specialized pro-resolving mediators” (SPMs) [9], will be summarized, reporting their biosynthetic pathways, the receptors on which they act in the known consolidated biochemical mechanisms, and those that are currently yet to be confirmed. A focus will be on studies on omega-3 fatty acid supplementation in sports, even if there are not yet unanimous indications. Despite this, we will provide indications for possible practical applications and future studies, which are undoubtedly necessary to clarify the omega-3 fatty acids used in sports practice.

## 2. Methods

The search was finalized in March 2024 with the search criteria (omega-3 fatty acids OR n-3 fatty acids OR fish oil) AND (sport OR sports OR performance) in PubMed as the database. We identified three macro areas of interest: the improvement of performance, the improvement of recovery after training or competitions, and the incidence of injuries or illnesses.

## 3. Biochemistry of Omega-3

**Figure 1:**
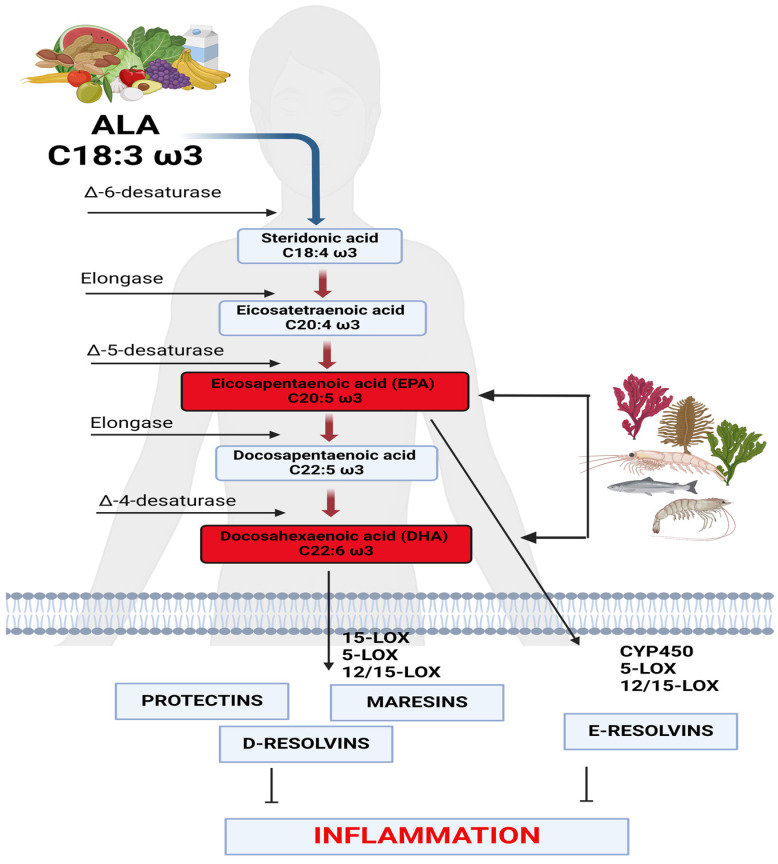
Biochemistry of omega-3. Utilization of ALA and synthesis of EPA and DHA. Created with BioRender (https://www.biorender.com/).

Omega-3 fatty acids are polyunsaturated fatty acids with the first double bond of the carbon chain present on the third carbon, counting from the last carbon atom (methyl) concerning the carboxylic group. Of this series, alpha-linolenic acid (ALA, 18:3 Ꙍ3) is the most important. ALA is an essential fatty acid, as our body is not capable of synthesizing it since animals, including humans, do not possess the Δ15-desaturase enzyme [10]. From ALA, eicosapentaenoic acid (EPA, 20:5 Ꙍ3) and docosahexaenoic acid (DHA, 22:6 Ꙍ3) can be synthesized via the combined activities of desaturase and elongase in the liver endoplasmic reticulum, following these steps: (i) ALA (18:3 Ꙍ3) ingested and absorbed from food is converted to stearidonic acid (18:4 Ꙍ3) by Δ6 desaturase and then (ii) can be elongated into eicosatetraenoic acid (20:4 Ꙍ3) and then (iii) desaturated again into EPA (20:5 Ꙍ3) by Δ5 desaturase. In these conversion steps, there is competition between EPA and the synthesis of arachidonic acid (ARA) (20:4 Ꙍ6) because the same enzymes are involved. It is thought that the rate-limiting step is the first enzyme, Δ6 desaturase. Moreover, both enzymes, Δ6 and Δ5 desaturases, are regulated by hormones, personal nutritional status (i.e., the amount of group B vitamins and vitamin C), and feedback inhibition by itself, creating a complex control network for endogenous synthesis by long-chain polyunsaturated fatty acids (PUFAs). In particular, the conversion to DHA (22:6 Ꙍ3) appears to be especially limited [10] (Figure 1).

However, this process is inefficient. It was reported in some studies that less than 8% of ALA is converted to EPA and less than 4% to DHA. As with other PUFAs, DHA and EPA are incorporated into the cell membrane phospholipids, regulating the cell membrane’s fluidity and permeability. Their fundamental role in the correct functioning of signal transduction in axons has emerged, and they are successfully used in the management of multiple sclerosis [11,12]. EPA is a precursor of other lipid mediators having anti-inflammatory and immunomodulatory biochemical action called the resolvins E series. Their synthesis is mediated by the isoenzymes 5-, 12-, and 15-lipoxygenases (LOXs). Similarly, from the DHA precursor, maresins and the resolvins D series are synthesized. PUFAs, in general, have also been established to be useful in improving reaction times in the normal population and athletes [13].

## 4. Nutritional Biochemistry: Sources and Supplements of Omega-3

Foods of plant origin, such as nuts and seeds, are rich in ALA, but as already underlined, despite being an essential fatty acid, it is not adequately converted into EPA and DHA. Foods that contain appreciable quantities of both EPA and DHA come from aquatic life. Therefore, several species of algae contain them (DHA is more present), but the amount of them per 100 g is relatively low [14]. Consequently, various species of fish (following the biomagnification effect) are a source of EPA and DHA, in particular, mackerel, sardines (therefore, oily fish), and salmon, and even crustaceans contain them. The quantity can reach a maximum of 3 g per 100 g of food. This amount should be taken in a daily quantity. Apart from some populations that base their diet on fish, such as the Inuit, these quantities are rarely reached; therefore, it is necessary to consider integration [15]. Fish liver oil has been part of Mediterranean culture since ancient Greece, as reported by Hippocrates, considered the father of modern medicine. Until 1800, cod liver oil was used to support growth and health. Even in Northern Europe, it was given to weak or disabled people [16]. The greatest concern regarding supplements based on fish oil is the presence of contaminants in seas, many of which are fat-soluble (dioxins and mercury, for example). To overcome the possible toxicity due to this, the first source used in supplements was krill, an almost microscopic shrimp present in plankton, which, being at the beginning of the alimentary chain, contains a lesser amount of contaminants. The cost of supplements derived from krill is quite high, but the benefits for human health are better as these microscopic shrimp are also a source of astaxanthin (an excellent antioxidant). For about 25 years, via molecular distillation, the supplement industry has made purified EPA plus DHA from fish oil. This process is operated in a vacuum, which allows the use of low boiling temperatures, concerning the atmospheric boiling point. It should be remembered that having various double bonds, EPA and DHA are particularly sensitive to temperature-induced peroxidation [17]. Therefore, a valid product should contain at least 50% EPA plus DHA. Today, it is possible to obtain effective omega-3 supplements from algae to satisfy the requests of vegetarians and vegans and, in any case, to have a product that, being at the base of the food pyramid, contains fewer contaminants [18].

## 5. Research on Omega-3: What Is Known and What Is Ongoing

For about twenty years since their discovery, specialized pro-resolving mediators (SPMs) have received a lot of attention from the scientific community. Currently, four distinct classes of fatty acid-derived mediators are recognized: (i) lipoxins, (ii) resolvins, (iii) protectins, and (iv) maresins.

Since lipoxins are derived from arachidonic acid (AA), they will be discussed briefly, as they are outside the focus of the present work. The other three classes are synthesized starting from EPA and DHA (in fact, the intermediate between the two, docosapentaenoic acid (DPA), is also a substrate for their synthesis). 

For convenience, hereafter, we refer to DHA and EPA instead of EPA and DHA, the right subsequence of their elongation.

**Figure 2:**
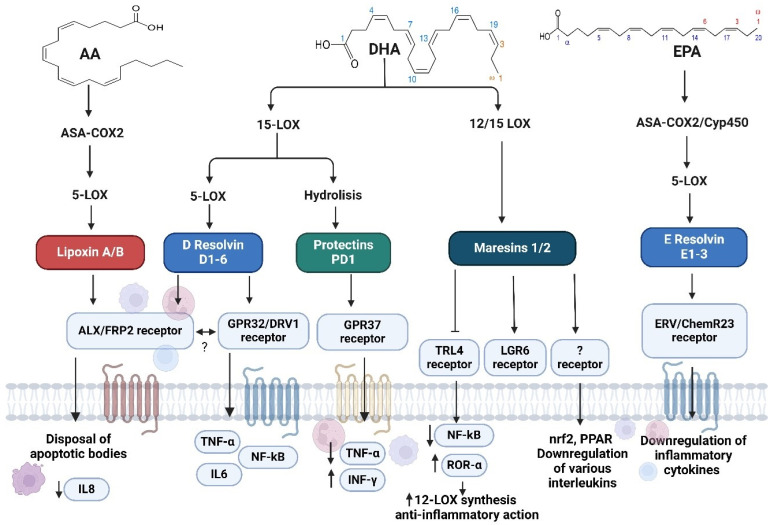
Precursors AA giving the lipoxin A/A (in red), DHA giving D Resolving D1-6 (in blue), Protectins (in light green) and Maresin (in dark green) and EPA giving E Resolving E1-3 (in blue). The fatty acids are converted via biosynthetic enzymes into SPMs, in turn activate their specific receptors to stimulate specific anti-inflammatory actions. Created with BioRender.

✓ **Lipoxins:** This class of anti-inflammatory molecules is synthesized by cyclooxygenase 2 (COX2) when it is bound to acetylsalicylic acid (ASA) in cooperation with lipoxygenase 5 (5-LOX). It is interesting to note how, normally, COX2 gives rise to pro-inflammatory prostaglandins such as PGF2, but the action of ASA shifts the synthesis toward an anti-inflammatory product. Lipoxin’s chemical structure is different as it is derived from an omega-6 fatty acid. It is present in two main forms, A and B, and both act through the lipoxin A receptor/formyl peptide receptor 2 (ALX/FPR2), a transmembrane receptor, expressed by almost all cells of the immune system. ALX/FPR2 promotes, among other things, the disposal of apoptotic bodies and the downregulation of IL8. It is also active in the regeneration of epithelia, particularly respiratory ones [19,20].
✓ **Resolvins D and E:** This is the group that has the greatest number of molecules, wherein two series are distinguished. Resolvins D are derived from DHA and have six different molecules. Their synthesis begins with the action of 15-LOX, and then epoxidation occurs, and finally, 5-LOX action yields them. Resolvins D act by the ALX/FPR2 and resolvin D receptor 1 (DRV1), also known as GPR32.
  This latter molecule needs lower concentrations of resolvin to be activated with respect to ALX/FPR2. The hypothesis is that, for example, resolvin D1 acts through DRV1 to maintain normal homeostasis. Only when the inflammation storm is more important is ALX/FPR2 activated. Resolvins’ action extends to various cells of the immune system, also downregulating the synthesis of inflammatory mediators, such as tumor necrosis factor-α (TNF-α) and IL6. Another relevant action concerns the modulation of NF-kB. The release of pro-inflammatory cytokines by macrophages is also downregulated [20,21].
  The resolvins E series derived from EPA have four molecules. Their synthesis starts with CYP450 or the COX2-ASA complex and then 5-LOX and epoxidation or reduction to give E1 or E2.
  Resolvins were the first molecules identified related to the resolution of inflammation, hence the name that highlights their action. The E series is probably the most studied of all, in particular, 1 and 2, which act through two receptors: the E series receptor (ERV), also called chemokine-like receptor 1 (CMKLR1), and chemerin receptor 23 (ChemR23). These receptors are not only expressed by cells of the immune system but also by dendrites and epithelial tissue. Their most marked action is on immune cells and also on the downregulation of inflammatory cytokines [20,22,23].
✓ **Protectins:** This group counts several molecules with differences in their stereochemistry and conjugations of carbon double bonds. They are derived by DHA and DPA, and all have three consecutive double bonds. Their synthesis begins with 15-LOX (the same pathway of resolvins D), but then they undergo epoxidation and, finally, enzymatic hydrolysis. They act via the receptor GPR37 [24,25]. It is interesting to note that the different protectins have different actions and modes depending on their affinity for the receptor itself. Protectins act by regulating the action of neutrophils and macrophages, as well as downregulating TNF-α and upregulating interferon-γ (IFNγ) [20,24,25].
✓ **Maresins:** Maresins represent the most recently discovered SPMs, i.e., in 2009. Their synthetic pathway varies as all synthesis begins through 12/15-LOX, and then soluble epoxide hydrolases 1 and 2 are obtained. Subsequently, the leukotriene C4 synthase and glutathione S-transferase MU 4 give MCTR1, the subsequent action of gamma-glutamyl transferase gives MCTR2, and, finally, a dipeptidase gives rise to MCTR3. This biological action is carried out by inhibiting the action of TLR4 with the consequent downregulation of NF-kB and the simultaneous activation of the RAR-related orphan receptor alpha (RORα), with the consequent upregulation of 12-LOX synthesis and anti-inflammatory action. LGR6 also seems to be the target of some maresins. Some studies have also shown actions on nrf2, PPARs, and the downregulation of various interleukins, but the mechanisms and receptors are still unknown [20,26,27,28] (Figure 2).

Several studies show that as a consequence of the use of DHA and EPA, there is an improvement in muscle protein synthesis (MPS) and, consequently, in muscle mass. At the moment, it is not clear whether this is due to the anti-inflammatory action of SPMs or whether there are other mechanisms involved [20,29,30]. Some studies show that following DHA and EPA supplementation, there is a greater incorporation of the two fatty acids into mitochondrial membranes, influencing the ADP/ATP ratio. Similarly, other reports highlight their incorporation into cell membranes, in particular, the sarcolemma. The hypothesis is that there is an influence on the fluidity of the membranes with a consequent optimization of the action of the transmembrane receptors (for example, those related to growth factors, such as insulin-like growth factor 1 (IGF1)) [31]. Another possible mechanism comes from a study carried out on pigs, where DHA and EPA supplementation led to an increase in the mRNA of the L-amino acid transporter (LAT-1) [32]. This transporter supervises, for example, the uptake of leucine, an amino acid noted to be an activator of the mechanistic target of rapamycin (mTOR), which, in turn, promotes protein synthesis. The greater presence of DHA and EPA in membranes could also decrease the availability of pro-inflammatory precursors, such as the omega-6 series (AA, for example).

An interesting study by Bhullar et al. [33] on cell lines demonstrated the involvement of DHA and EPA in three important factors that regulate the differentiation of muscle stem cells: paired box 7 (Pax7), myogenic differentiation 1 protein, and myogenin. If this were to be confirmed in vivo, it would be direct evidence of a mechanism different from that linked to the anti-inflammatory state.

In another study, Lee and co-workers [34] highlighted the activation by DHA and EPA of the peroxisome proliferator-activated receptor gamma co-activator 1-alpha (PGC-1α), a cellular mediator of considerable importance for muscles as it is anti-inflammatory but also responsible for mitochondrial biogenesis. The experiments, however, were carried out on cell lines.

Finally, an effect that could have an impact on sports practice is the beneficial action of DHA and EPA in the nervous system, in particular, on cognitive function. It is known that DHA and EPA are necessary, starting from gestation, for the correct development of the entire nervous system, in particular, the central one (the brain). This, as already underlined, is due to the presence of the two fatty acids in the cell membranes of the nervous system; in particular, the conduction of nervous impulses along the neurons seems to be influenced by their presence [35]. In this view, Godos and co-workers suggested in a meta-analysis that fish consumption is associated with a lower risk of cognitive impairment/decline in a dose–response manner [36].

## 6. Use and Benefit in Sport: Practical Suggestions

Consequently, based on the studies presented, the main biochemical mechanism of DHA and EPA seems to be linked to their anti-inflammatory activity. We report in Table 1 the outcomes and the possible scientific rationale for their use, the context in which to use them, and a general evaluation.

Taking together all the results from this review, our recommendations are the following:

❖ Attention should be given to the manufacturing of omega-3 products; not all supplements are the same [37]. It is better to choose one with a percentage of at least 50% DHA and EPA per soft gel or capsule; this should guarantee that they have undergone vacuum distillation.
❖ Do not consider omega-3 commercial proposals that contain ALA. As underlined, it is poorly effective in providing DHA and EPA.
❖ Consider at least 1.5 g of DHA plus EPA daily, but the dose could be increased or even doubled in sports that can evoke an inflammatory state (e.g., endurance and ultra-endurance sports and heavy weightlifting) or that regularly involve trauma (e.g., combat sports and rugby).
❖ No particular timing of administration for omega-3 is mandatory.
❖ Evaluate the daily intake according to a food diary and adjust the dosage of omega-3 accordingly.
❖ Evaluate subjective sensitivity, possibly considering omega-3 gastro-resistant preparations.
❖ There are valid supplements of omega-3 obtained from algae, suitable for vegetarian or vegan athletes, even if they normally have a lower percentage of DHA and EPA.

## 7. Discussion

We selected 53 studies, and the data from them were very variable in the duration of supplementation (excluding acute studies using a single dose), spanning from 2 to 24 weeks. The studies had other interesting considerations. They were carried out more on amateurs than elite athletes, probably because it is more difficult to operate on professional athletes than on amateurs. There is some reluctance regarding the use of omega-3, even if the reported intake is sub-optimal [38]. Another point difference between the studies was in the dosage of DHA plus EPA (from 0.1 to 6 g per day). The timing was rarely considered, just as the source and production methods were often communicated but in a generic manner. The studies analyzed, in which omega-3 supplementation was used, regarded the following sports: soccer, football, rugby, taekwondo, judo, padel, cycling, track and field, running, and long-distance running. Omega-3 was used for several reasons. This was an obvious problem that was linked to the discrepancy in the tests carried out to evaluate the possible improvements in performance, for example, heart rate, blood pressure, performance at exhaustion, VO_2_ max, power and peak output, countermovement jump, perceived soreness, plasma metabolites or molecules (creatinine kinase, C-reactive protein, triglycerides, interleukins, and COX1 and -2 mRNA). An aspect that has not been considered is that of an estimate of the intake of omega-3 from the diet. In some studies, the omega-3 index was evaluated, which consists of the analysis of the quantity of omega-3 in the cell membranes of erythrocytes, but it is still controversial whether this is a truly reliable index of the quantity of DHA and EPA in the entire organism [38,39,40,41]. In a randomized study on footballers, Gravina et al. [42] highlighted an improvement in aerobic capacity following 4 weeks of omega-3 supplementation. Black’s group showed a moderate but significant improvement in muscle pain, which translated into improved explosive power. This effect could be explained thanks to the anti-inflammatory action. In this case, the dosage was 2 g per day of DHA and EPA. The studies by Martorell et al. [43] showed how supplementation with 1.14 g of DHA provides support in improving the anti-inflammatory and antioxidant state induced by intense exercise in athletes. A similar result was obtained by Kolar et al. [44], supplying omega-3 through eggs enriched with PUFAs. In this case, the result was valid, but the actual dosage was difficult to establish. Thielecke et al. [45] showed how DHA plus EPA produced a decrease in various inflammatory markers in high-level athletes. The protocol of Philpott et al. [46] involved the addition of 1.1 g of DHA plus EPA to a shake normally taken post-workout. The intervention produced a decrease in creatine kinase and post-exercise muscle pain in professional footballers. In the study carried out by Jaworska et al., a higher dosage was used, i.e., 2.4 g per day in three doses for 3 weeks. The subjects were high-level long-distance runners. Evaluating the 24 subjects, an improvement in inflammatory mediators following intense exercise was noted. A study with a different outcome from the others was that by Guzman et al. [47], in which 24 professional footballers were supplemented with 3.4 g of DHA per day for four weeks, also evaluating a seven-day food diary. This produced an improvement in neuromotor skills, particularly in neural RT [13]. Regarding applications of amateur physical exercise, there are more studies with better outcomes overall, but this may also be due to the lower training level. Good results have also been obtained in the management of sarcopenia, combining physical exercise and omega-3, as an inflammatory component is most likely part of the sarcopenia condition and type 1 diabetes [48,49]. Overall, it can be said that there is a strong scientific rationale for recommending the additional intake of DHA and EPA for athletes, especially elite ones. This could be of even more interest if the athletes have pathological conditions. In two of our case reports, we showed how two high-level athletes (swimming and vovinam) with different pathologies (celiac disease and type I diabetes) benefited from supplementation with 1.5 g of DHA and EPA daily [50,51]. A particular context that concerns the use of DHA and EPA is that of combat sports and, in general, where concussions are normal sporting practice and not accidental, in particular, regarding the maintenance of neuronal plasticity following blows to the head; however, its use has not yet been confirmed by scientific studies [50,51,52,53]. The following limitations and needs were found in this review: (i) Studies should be organized more coherently in the ambit of sports and physical activity, evaluating the anti-inflammatory effects first through cytokines, gene expression, and perhaps microRNA. This is important, particularly in sports where inflammation plays an important role. (ii) The daily intake from foods of omega-3 should be evaluated in order to define an adequate dosage. (iii) Finally, performance outcomes should be evaluated uniformly. For example, attention should be paid to the tests to be used based on the expected result, using them to calm inflammation due to endurance. It is not guaranteed that this will translate into an improvement in the VO_2_ max but may, instead, translate into a lower incidence of respiratory syndrome.

## 8. Conclusions and Future Directions

The anti-inflammatory action of the two omega-3 fatty acids DHA and EPA is well established, even if unrelated to sports, solidifying the scientific rationale that justifies their integration. There are food sources available, in particular, blue fish and salmon, but to obtain appreciable quantities of them, one would have to eat quantities which, for many, are unacceptable. Other pathways of action have been hypothesized, which have shown evidence in vitro but have yet to be confirmed in vivo. The studies on sports have conflicting results; there are various that verify the effectiveness of DHA and EPA. It remains to be defined whether we want to consider these supplements as directly ergogenic (i.e., capable of directly improving performance) or as supplements that put the athlete in the best health conditions and, therefore, have a positive influence on performance.

## Figures and Tables

**Table 1:** Summary of possible applications of DHA and EPA supplementation.

Sports/Physical Activity Context	Rating	Possible Biochemical Mechanism/Signaling Activation
Strength training	5	mTOR activation/protein synthesis
Endurance	8	Airway syndrome/flu mitigation Free radicals and inflammation modulation
Mixed (team sports)	7	Reduced post-workout soreness or DOMS
Immobilization	7	Prevention of muscle wasting (inflammation modulation)
Combat sports/concussion	7	Possible support for neuron plasticity and functional preservation

Rating resulting from science: 0—poor; 10—strong.

## Abbreviations

**ADP**	Adenosine diphosphate
**ALA**	Alpha-linolenic acid
**ALX/FPR2**	Lipoxin A receptor/formyl peptide receptor 2
**ARA**	Arachidonic acid
**ASA**	Acetylsalicylic acid
**ATP**	Adenosine triphosphate
**EPA**	Eicosapentaenoic acid
**ChemR23**	Chemerin receptor 23
**CMKLR1**	Chemokine-like receptor 1
**COX1**	Cyclooxygenase 1
**COX2**	Cyclooxygenase 2
**CYP450**	Cytochromes P450
**DHA**	Docosahexaenoic acid
**DPA**	Docosapentaenoic acid
**DOMS**	Delayed onset muscle soreness
**DRV1**	Resolvin D receptor 1
**ERV**	E series receptor
**FOR**	Functional overreaching
**GPR32**	G protein-coupled receptor 32
**GPR37**	G protein-coupled receptor 37
**IFNγ**	Interferon-γ
**IGF1**	Insulin-like growth factor 1
**IL6**	Interleukin 6
**IL8**	Interleukin 8
**NF-kB**	Nuclear factor kappa-light-chain-enhancer of activated B cells
**LAT-1**	L-amino acid transporter
**LGR6**	Leucine-rich repeat-containing G protein-coupled receptor 6
**MCTR1**	Maresin conjugates in tissue regeneration 1
**MCTR2**	Maresin conjugates in tissue regeneration 2
**MCTR3**	Maresin conjugates in tissue regeneration 3
**MPS**	Muscle protein synthesis
**mTOR**	Mechanistic target of rapamycin
**nrf2**	Nuclear factor erythroid 2-like 2
**ONS**	Oral nutritional supplement
**Pax7**	Paired box 7
**PGC-1α**	Peroxisome proliferator-activated receptor gamma co-activator 1-alpha
**PGF2**	Prostaglandin 2
**PPARs**	Peroxisome proliferator-activated receptors
**PUFAs**	Polyunsaturated fatty acids
**RORα**	RAR-related orphan receptor alpha
**RT**	Reaction time
**SPMs**	Specialized pro-resolving mediators
**TLR4**	Toll-like receptor 4
**TNF-α**	Tumor necrosis factor α
**VO_2_ max**	Maximal oxygen consumption
**5-LOX**	Lipoxygenase 5
**12-LOX**	Lipoxygenase 12
**15-LOX**	Lipoxygenase 15
